# Recovery attributions and future expectations for antibiotics after precautionary prescribing

**DOI:** 10.1093/eurpub/ckaf146

**Published:** 2025-08-26

**Authors:** Elisabeth D C Sievert, Lars Korn, Rian Gross, Robert Böhm, Cornelia Betsch

**Affiliations:** Health Communication, Institute for Planetary Health Behaviour, University of Erfurt, Erfurt, Germany; Health Communication, Implementation Research, Bernhard Nocht Institute for Tropical Medicine, Hamburg, Germany; Health Communication, Institute for Planetary Health Behaviour, University of Erfurt, Erfurt, Germany; Health Communication, Implementation Research, Bernhard Nocht Institute for Tropical Medicine, Hamburg, Germany; Department of Occupational, Economic, and Social Psychology, Faculty of Psychology, University of Vienna, Vienna, Austria; Department of Occupational, Economic, and Social Psychology, Faculty of Psychology, University of Vienna, Vienna, Austria; Department of Psychology and Copenhagen Center for Social Data Science, University of Copenhagen, Copenhagen, Denmark; Department of Banking and Finance, University of Innsbruck, Innsbruck, Austria; Health Communication, Institute for Planetary Health Behaviour, University of Erfurt, Erfurt, Germany; Health Communication, Implementation Research, Bernhard Nocht Institute for Tropical Medicine, Hamburg, Germany

## Abstract

Antibiotic overuse is a significant public health challenge, and antibiotics are often prescribed as a precaution. While precautionary prescribing often aims to meet perceived patient expectations, it can unintentionally reinforce the belief that antibiotics are necessary and effective. This study examines how the communication of precautionary antibiotic prescribing shapes patients’ beliefs in what actually made them better and how these causal beliefs, in turn, affect their expectations for antibiotics in future illness situations. In the two preregistered online experiments with UK adults (*N *= 252 and *N *= 2448), participants imagined having an ear infection, receiving a precautionary prescription, taking antibiotics, and recovering shortly thereafter. We manipulated whether participants received statistical information about the likelihood of a self-limiting recovery (base-rate information) before the prescription. Participants then evaluated what caused their recovery and whether they expected to receive antibiotics for a future, unrelated illness. Across both studies, participants generally attributed their recovery to the antibiotics, even when told that the infection was likely to resolve on its own. This attribution was associated with stronger expectations of receiving antibiotics for future illnesses. However, participants who received base-rate information more accurately attributed their recovery, suggesting that statistical context can partially correct causal beliefs. Precautionary prescribing can unintentionally shape patients’ beliefs about antibiotic efficacy, which, in turn, reinforces future demand. Providing patients with brief information on self-limiting infections may help reduce inappropriate antibiotic expectations and support prudent and more sustainable prescribing practices.

## Introduction

Antibiotics are essential to modern medicine, but their overuse is a pressing public health concern [1]. One driver of this problem is the prescription of antibiotics for self-limiting conditions. For instance, in the UK, an estimated 23% of all antibiotic prescriptions are inappropriate [2], with acute otitis media being among the leading diagnoses associated with unnecessary antibiotic use [2]. Some overprescriptions stem from clinical uncertainty, but patient expectations are also influential. Several studies show that a patient’s expectation of receiving antibiotics causally influences antibiotic prescribing [3–5]. Patients’ expectations do not develop in isolation: physicians do not always communicate clearly about the limitations and appropriate use of antibiotics [6, 7]. When such information is lacking, patients may assume that antibiotics are necessary [8, 9]. Moreover, even when not medically indicated, physicians may prescribe antibiotics to preserve patient relationships or avoid conflict [10, 11]. This suggests a bidirectional problem in which inadequate physician communication and patient expectations reinforce each other, increasing the likelihood of potentially unnecessary prescriptions.

This practice may unintentionally reinforce the expectations it seeks to satisfy. When antibiotics are prescribed as a precaution, and the patient subsequently recovers, the patient may infer that the antibiotics caused the improvement—regardless of whether the infection was self-limiting. Over time, such causal attributions may increase the perceived necessity of antibiotics, reinforcing a cycle of demand and overprescription.

This study investigates whether precautionary antibiotic prescriptions lead patients to falsely attribute their recovery to antibiotic use, fostering inappropriate expectations for future antibiotic treatment. Drawing on attribution theory [12, 13], we assess whether the perceived locus of causality, attributing recovery to internal factors (the immune system) or to external ones (antibiotics), is shaped by the way the precautionary prescription is communicated. Specifically, we assess whether base-rate information about the self-limiting nature of infections can correct potentially false inferences. Base-rate information is a form of statistical context that may help patients distinguish correlation from causation [14, 15], thereby weakening potentially erroneous attributions to antibiotics. By manipulating whether participants received base-rate information alongside a precautionary prescription, we explore whether such information reduces the attribution of the recovery to antibiotics. Additionally, we are interested in understanding whether the attribution formed in one context (e.g., otitis media) generalizes into expectations for antibiotics for another, unrelated illness scenario (e.g., upper respiratory tract infection). The two illness contexts are intentionally distinct to avoid simple associative learning effects and investigate whether broader beliefs about antibiotic necessity carry over across situations, based on assumptions by attribution theory [12, 13] that individuals develop causal models in the absence of clear contextual boundaries.

## Study 1

To investigate how precautionary prescribing of antibiotics and base-rate information affect patients’ causal beliefs and expectations, we formulated four preregistered hypotheses. First, we tested whether base-rate information influences how people interpret their recovery. Specifically, we hypothesized that participants who receive high base-rate information—indicating that the infection was likely to be self-limiting—will be less likely to attribute their recovery to antibiotics compared to participants in the control group without base-rate information (H1a). In contrast, we expected that participants in the low base-rate condition—suggesting that self-limiting infections are unlikely—will show stronger attribution to antibiotics than the control group without base rate information (H1b), and also a stronger attribution than participants in the high base-rate condition (H1c). Second, we expected that a higher recovery attribution to antibiotics would be associated with higher expectations of receiving antibiotics for a subsequent, unrelated illness (H2), reflecting a potential spillover effect from one illness context to another. We preregistered this association to be tested across the full sample, with exploratory analyses examining whether the strength of the association varied by condition.

### Methods

In April 2024, we conducted a preregistered experimental online survey with *N* = 252 participants from the United Kingdom, recruited via Prolific. The sample size was based on an *a priori* power analysis using G*Power for a one-way ANOVA (*f* = 0.25, α = 0.05, power = 0.95). The participants received a remuneration of £0.80 for completing the 6-min survey. Ethical approval was granted by the University of Vienna (2024/W/010).

The participants read a vignette describing a hypothetical scenario of a doctor’s visit for an ear infection, during which the doctor told them that their symptoms were unspecific and that the infection could be self-limiting (see OSF). In the experimental conditions, the doctor provided base rate information about the likelihood of self-limitation before offering antibiotics: either 85% (high base rate), 15% (low base rate), or the same clinical explanation without base rate information in the control condition. In the high base-rate condition, the doctor explained that this indicated a strong likelihood that the infection would be self-limiting, meaning that antibiotics would likely not be necessary to help the symptoms. In contrast, in the low base-rate condition, the doctor suggested that the infection was unlikely to resolve on its own, making antibiotic treatment likely to be needed. The high base rate was based on estimates suggesting that ∼85% of acute otitis media infections do not require antibiotic treatment [16]. For the low base rate, we used the remaining percentage. In all conditions, the doctor concluded by offering antibiotics as a precaution, but emphasized it was up to the patient whether to take them. Then, participants were told that they had taken the antibiotics and recovered after a few days. They then indicated the extent to which they attributed their recovery to the antibiotics versus their immune system using a rating scale ranging from 0 (*immune system*) to 100 (*antibiotics*). This was followed by a scenario of an unrelated illness (upper respiratory tract infection) occurring a few months later, for which the participants indicated their expectations of receiving antibiotics via the mean of six items on a Likert scale ranging from 1 (*strongly disagree*) to 7 (*strongly agree*). The items included statements such as ‘Antibiotics will be effective in treating my infection’ and ‘I would request an antibiotic prescription from my doctor’ (Cronbach’s α = 0.94) [17, 18].

Due to non-normality of the attribution variable (*W *= 0.92, *P* < .001), we deviated from the preregistration and conducted a nonparametric Kruskal–Wallis test to assess the influence of base-rate information on participants’ attribution of the recovery to antibiotics, followed by *post hoc* comparisons using the Wilcoxon rank-sum test (H1a-c).

To assess the relationship between the attribution of the recovery to antibiotics and subsequent expectations for antibiotics (H2), we calculate Spearman correlation coefficients of these variables across the full sample and then exploratively stratified by condition.

### Results

The participants were aged 19–83 years (*M *= 43 years, SD = 15). Among them, 50% were women, and 54% had a bachelor’s degree or higher (overview of the participants’ characteristics [Table S1] in Supplementary Materials).

**Figure 1.**
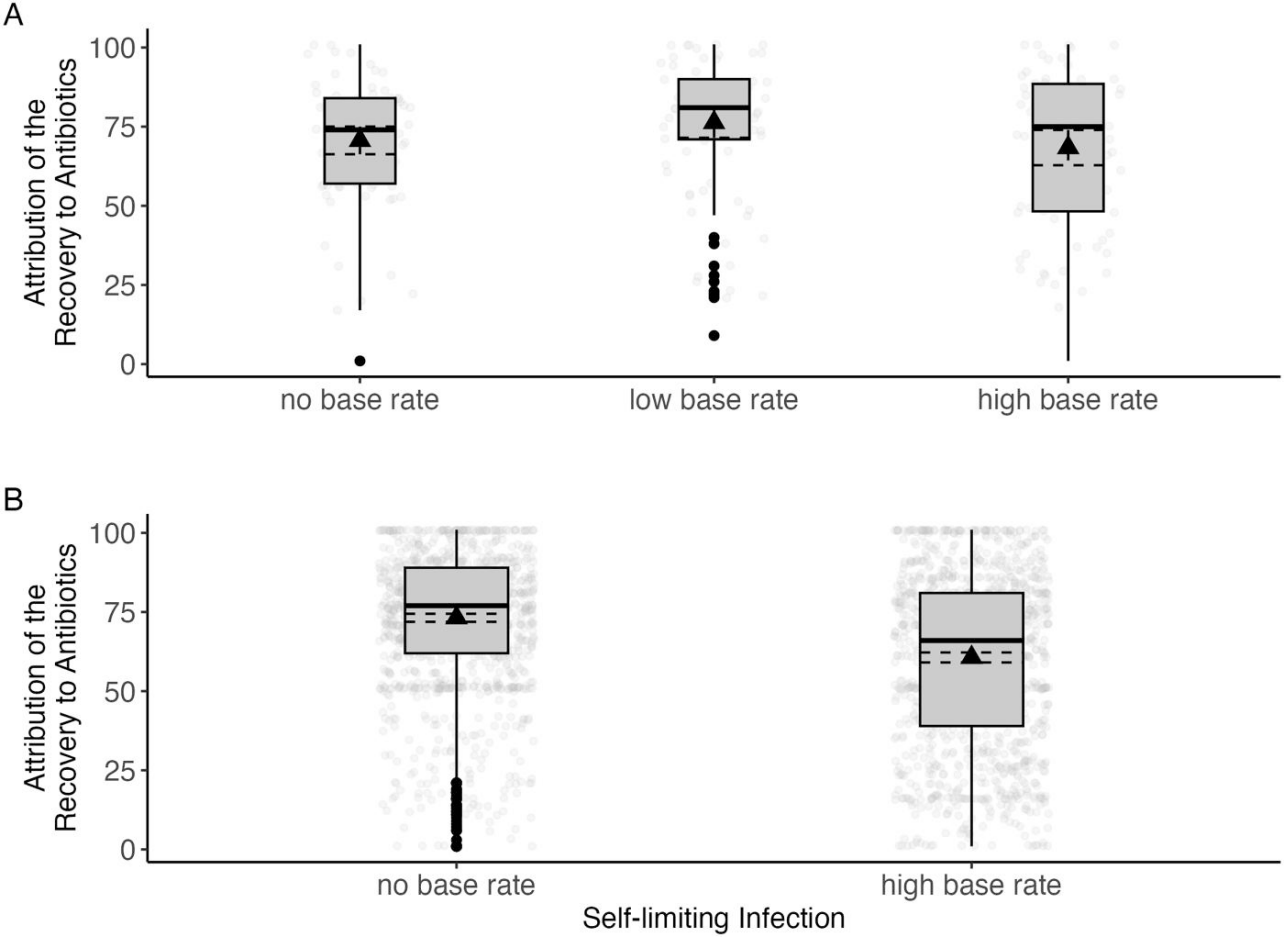
Attribution of the recovery to antibiotics by experimental condition in Study 1 (A) and Study 2 (B). Higher scores indicate a higher attribution to antibiotics. Box plots display the median, interquartile range, mean (black triangle), and jittered individual responses depending on base-rate information for self-limiting infections. In Study 1, attribution was highest in the low base-rate condition, but no significant difference was found between the high base-rate condition and control condition. In Study 2, attribution was significantly lower in the high base-rate condition compared to control.

As shown in Fig. 1A, the attribution of recovery to antibiotics was quite strong, but there were differences between the conditions, χ^2^(2) = 6.53, *P* = .038, ε^2^ = 0.02. In line with H1b, *post hoc* comparisons revealed that the participants in the low base-rate condition were more inclined to attribute their recovery to antibiotics than those in the control group, *P* = .044, *d *= 0.38. No other single comparison was significant (low vs. high base rate: *P* = .149, *d *= 0.31; high base rate vs. control: *P* = .927, *d *= 0.01).

**Figure 2.**
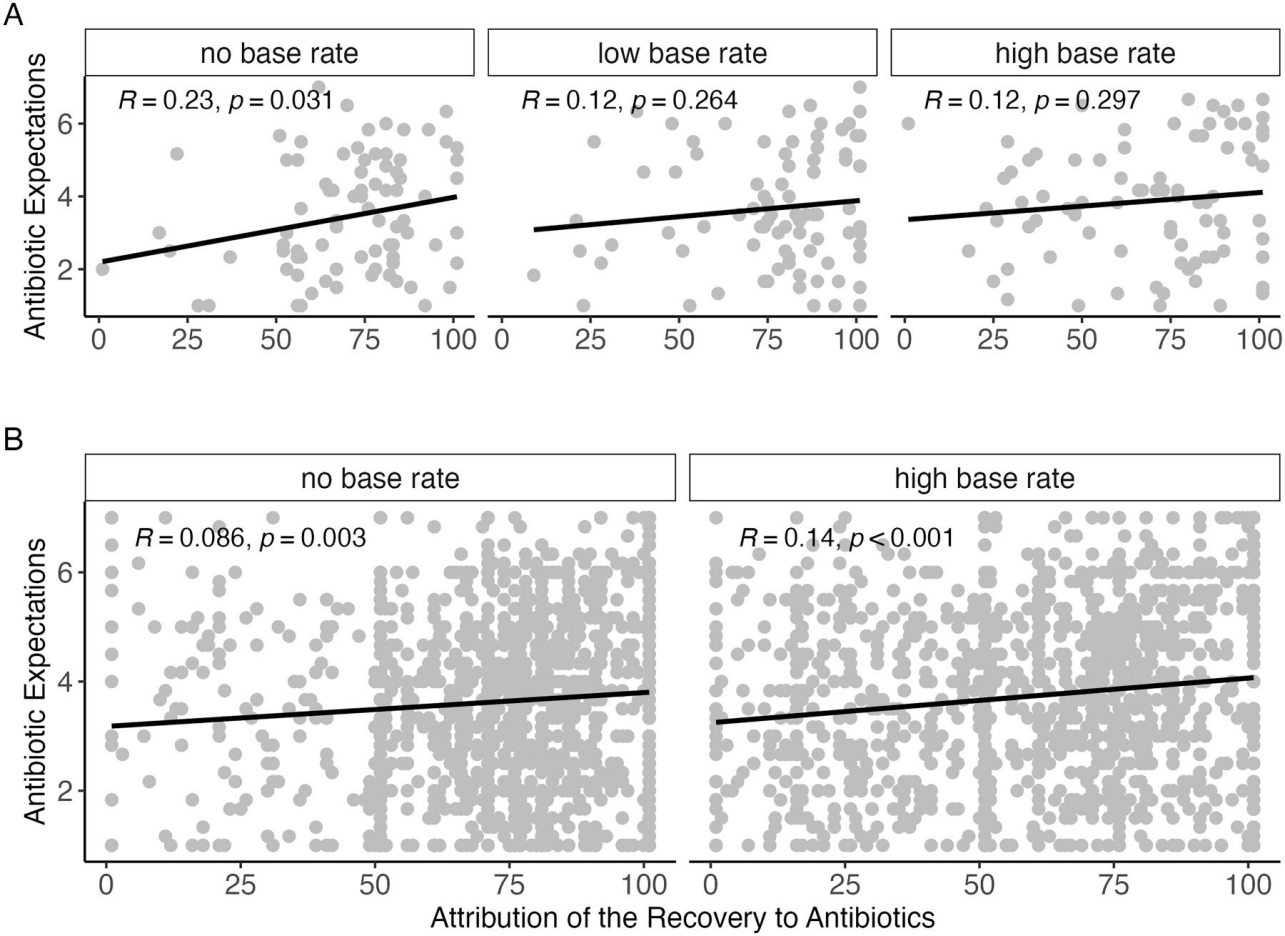
Correlation between recovery attributions and expectations to receive antibiotics in an unrelated second situation by experimental condition in Study 1 (A) and Study 2 (B). Higher scores indicate a higher attribution to antibiotics and higher expectations of receiving antibiotics. Regression lines indicate linear trends; *R* and *P* values reflect correlation coefficients. While Study 1 suggested that recovery attributions spill over primarily in the absence of base-rate information, Study 2 found a similar link between recovery attribution and future expectations, indicating generalized effects regardless of statistical context.

When participants attributed their recovery more strongly to antibiotics, they also reported higher expectations to receive antibiotics for the subsequent illness, *r*(250) = 0.14, *P* = .027, consistent with H2. As Fig. 2A shows, when stratifying this association by condition, we only found a significant association in the control group that had not received any base-rate information, *r*(83) = 0.23, *P* = .031, but not in the low base-rate, *r*(83) = 0.12, *P* = .264, and high base-rate conditions, *r*(80) = 0.12, *P* = .297.

### Discussion

In Study 1, base-rate information only partially affected participants’ attribution of their recovery to antibiotics. In the high base-rate condition, where participants were told that their infection was likely self-limiting, attributions remained high. One potential explanation is that the act of prescribing antibiotics as a precaution reinforced pre-existing beliefs about their efficacy, thereby overriding the statistical information. This is consistent with prior research showing that people often neglect or underuse base-rate information, especially when it conflicts with intuitive assumptions [14, 15]. In contrast, the base-rate manipulation was more effective in the low base-rate condition, where it strengthened the belief that antibiotics were needed. Another explanation possibly lies in the wording of the manipulation itself: the explanation that antibiotics may not be necessary could have been too vague or implicit to counteract general beliefs about the usefulness of antibiotics. Participants might have interpreted this phrasing as such that antibiotics were still beneficial for recovery (for instance, to shorten the length of the infection), even if not strictly essential.

These strong attributions had spillover effects: participants who more strongly attributed their recovery to antibiotics also reported higher expectations of receiving antibiotics for a future, unrelated illness. Notably, this attribution–expectation link was significantly stronger in the control condition than in the base-rate conditions. Based on attribution theory [12, 13], people rely on contextual cues to make sense of outcomes. Participants who received base-rate information may have treated it as specific to the first illness, limiting its generalization to future scenarios, whereas those in the control condition, who were lacking such cues, may have formed broader causal beliefs that shaped expectations for subsequent treatment. To assess the robustness of these findings, we conducted a partial replication of the first study.

## Study 2

The aim of Study 2 was to replicate the attribution-expectation link with greater statistical power and to more precisely investigate how base-rate information influences recovery attributions in contexts where antibiotics are likely unnecessary. This time, the wording of the vignette was more explicit (‘antibiotics are ineffective in this case’), and mentioned trade-offs in terms of side effects to see whether this phrasing would be more effective to challenge pre-existing beliefs about antibiotics than the cautious phrasing in Study 1 that antibiotics may not be necessary.

We expected that participants who receive high base-rate information—indicating that the infection was likely self-limiting—would attribute their recovery less strongly to antibiotics than participants in the control condition, who received no base-rate information (H1). We also predicted that participants who more strongly attributed their recovery to antibiotics would have higher antibiotic expectations in the future (H2). Finally, we hypothesized that the attribution–expectation link would be stronger in the control condition than in the high base-rate condition (H3), as the absence of contextual cues in the control group would lead participants to generalize their beliefs about the necessity of antibiotics more broadly.

### Methods

We conducted a preregistered experimental online survey with *N* = 2448 participants (based on an *a priori* power analysis with G*Power for a one-tailed z-test comparing two independent Pearson correlations; *q* = 0.01, α = 0.05, power = 0.80) from the UK, recruited via Prolific in April 2025. The participants received a remuneration of £0.73 for completing the 5-minute survey. Ethical approval was granted by the University of Vienna (2025/W/009).

In Study 2, participants were presented with a slightly adapted version of the hypothetical scenario from Study 1, involving a doctor’s visit for an ear infection. After being told they had taken antibiotics and recovered, they rated how much they attributed their recovery to their immune system versus antibiotics (0 = immune system, 100 = antibiotics). As in Study 1, this was followed by a second scenario of a different illness (upper respiratory tract infection), in which participants indicated their expectations of receiving antibiotics using the six items used in Study 1 (Cronbach’s α = 0.95).

In contrast to Study 1, which included three experimental conditions, Study 2 focused only on the high base-rate and a control condition without base-rate information. We dropped the low base-rate condition to focus on the most relevant comparison: the high base rate group is particularly important because it reflects situations where antibiotics are very likely unnecessary, yet in Study 1, participants in this condition still showed relatively high recovery attributions to antibiotics. This disconnect between objective information and subjective attribution is important for understanding how precautionary prescribing may reinforce false beliefs about antibiotic effectiveness. The control group received no base-rate cues, making it structurally comparable with the control group in Study 1. We also added an encoding check to ensure that participants processed the base-rate information as intended; they were asked to indicate the percentage of infections that resolve without antibiotics, or, in the control condition, to report that no such information had been provided.

To test whether base-rate information influenced recovery attributions (H1), we conducted an independent sample t-test comparing attribution scores between the high base-rate and no base-rate (control) condition. To assess whether higher recovery attribution after the first scenario was associated with increased expectations of receiving antibiotics in a future scenario (H2), we computed a Pearson correlation across the full sample, examining the relationship between attribution scores from the first scenario and antibiotic expectations in the second. To test whether this association differed depending on the presence or absence of base-rate information (H3), we stratified the correlation analysis by condition and calculated separate Pearson correlation coefficients for each group, which were then statistically compared using a Fisher z-test for independent correlations.

### Results

The participants were aged 18–87 years (*M *= 42 years, SD = 14). Among them, 48% were women, and 61% had a bachelor’s degree or higher (overview [Table S2] in Supplementary Materials).

Similar to Study 1, attribution of the recovery to antibiotics was quite strong, but participants in the high base-rate condition attributed their recovery less to antibiotics (*M *= 60.65, SD = 28.01) than those in the control condition (*M *= 73.20, SD = 22.47), *t*(2349.8) = –12.24, *P* < .001, *d* = –0.49 (Fig. 1B), supporting H1.

There was a small positive correlation between participants’ attribution of their recovery to antibiotics and their expectations of receiving antibiotics in a future illness scenario, *r*(2446) = 0.11, *P* < 0.001, which suggests that the more strongly individuals believed that antibiotics were responsible for their recovery, the more likely they were to expect antibiotics in a future situation. This yields evidence in favor of H2. The correlation between recovery attribution and antibiotic expectations was *r*(1213) = 0.09, *P* = .003 in the control condition and *r*(1231) = 0.14, *P* <.001 in the high base-rate condition. A one-tailed Fisher z-test indicated that the difference between these correlations was not statistically significant, *z* = –1.42, *P* = .922, contradicting H3; see Fig. 2B.

The encoding check was answered correctly by 84.5% of the participants. Our results reported above remained qualitatively the same when excluding participants who answered the encoding check incorrectly (see Supplementary Materials).

### Discussion

In Study 2, we found that providing high base-rate information led participants to attribute their recovery less to antibiotics than those in the control group, consistent with the first hypothesis. This suggests that base-rate information can correct recovery attributions, aligning them more with the clinical context. However, attribution levels remained high, suggesting that the prescription itself may act as a strong cue for perceived efficacy, potentially overriding the corrective effects of the statistical information. Again, we observed a small positive association in that participants who attributed their recovery more strongly to antibiotics were more likely to expect antibiotics for a future infection, consistent with our second hypothesis. This reflects a spillover effect: individuals who believe that antibiotics were responsible for their recovery are more likely to expect them in subsequent, unrelated illnesses. In this study, contrary to our third hypothesis, the strength of the attribution–expectation association did not differ significantly between the base rate and control conditions, despite the increased statistical power. While this seems to contrast with Study 1, where a significant association was observed only in the control group, the effect sizes were small and similar in magnitude across studies. This pattern suggests that the statistical significance observed between the stratified correlations of recovery attribution and antibiotic expectations in Study 2 likely reflects its larger sample size rather than a substantial difference in the strength of the association. These findings indicate that base-rate information does not appear to meaningfully alter how strongly recovery attributions relate to future expectations for antibiotics. Based on attribution theory [12, 13], we had reasoned that participants who received statistical information might treat their recovery as context-specific, thereby limiting generalization to new situations. In contrast, we expected participants in the control condition, who were lacking such contextual cues, to form broader, more generalized causal beliefs. The absence of this pattern in Study 2 suggests that base-rate information may not always function as a boundary cue in the way attribution theory suggests, which aligns with research indicating that individuals neglect or underweight base-rate information, especially when it conflicts with salient cues or intuitive beliefs [14, 15, 19, 20].

## General discussion

Taken together, the two studies offer evidence that precautionary antibiotic prescribing can shape patients’ causal beliefs, and these, in turn, influence their expectations of receiving antibiotics in future medical situations. Across both studies, participants tended to strongly attribute their recovery to antibiotics, even when told that their infection was likely to be self-limiting. This suggests that precautionary prescriptions may reinforce the potentially erroneous belief that antibiotics were effective, which can increase future demand.

On a more positive note, findings from both studies indicate that providing statistical information about the likelihood of an infection being self-limiting can affect participants’ attribution of their recovery to antibiotics. This suggests that even brief base-rate information can help recalibrate patients’ beliefs about treatment effectiveness and improve causal reasoning. However, the absolute level of attributing recovery to the antibiotics remained relatively high even when participants were informed that most similar infections resolve without antibiotics. This effect may be explained by merely receiving a prescription, which likely served as a strong behavioral cue that antibiotics were necessary. When verbal information about self-limitation contradicts clinical behavior (e.g., prescribing antibiotics), people may rely more on the doctor’s action than their words, which reduces the impact of corrective information. An alternative explanation for the high attribution is that participants may have attributed their recovery to the act of taking antibiotics because it is a concrete behavior. Research suggests that individuals often exhibit an action bias, i.e., a preference for attributing outcomes to actions rather than inaction, especially in health-related decisions [21–23], including antibiotic use [24, 25]. Therefore, recovery may have been perceived as more causally linked to the active treatment (i.e., taking antibiotics) than to the passive process, i.e., a response by one’s immune system.

Across both studies, stronger recovery attributions to antibiotics were linked to higher expectations of receiving antibiotics for a subsequent, unrelated illness. This spillover effect highlights how beliefs shaped by precautionary prescribing can generalize across contexts and reinforce future demand. Although only a small effect, this can contribute to unnecessary antibiotic use over time when false recovery attributions from past illnesses lead to more use in future situations.

We did not find consistent evidence that base-rate information alters this spillover effect. Base-rate information may be a relatively weak behavioral cue, especially when it competes with salient behavioral signals (receiving a prescription) which are also connected to strong social norms (do what your doctor says). In addition, base-rates are inherently hard to process and base-rate neglect is a common phenomenon in decision research [14, 15, 19, 20]. Participants may have focused more on the fact that they were told they took antibiotics than on the probabilistic framing, leading to similar inferences across conditions. Building on these findings, future research should examine the formation of recovery attributions through a broader lens. For example, it would be informative to assess further attribution dimensions as proposed by attribution theory. Future research could extend this by also assessing other attribution dimensions, such as stability and controllability [12, 13], to examine whether individuals perceive antibiotic effectiveness as a consistent and manageable factor across illness contexts. Research in other health domains has shown that attributional patterns are shaped by various factors, including symptom visibility, perceived controllability, and cultural beliefs [26–28]. This suggests that recovery attributions are not only constructed cognitively but are also socially and contextually shaped. Applying these insights to infections may help clarify how individuals form causal beliefs about antibiotic treatment efficacy, and why simple interventions such as base-rate information may not be sufficient on their own.

A limitation of this study is that participants responded to hypothetical illness scenarios rather than experiencing real symptoms. Future research should aim to replicate these findings in clinical settings. In addition, accumulator-based models could offer a promising avenue for examining how expectations and causal attributions evolve across repeated decisions [29, 30], potentially identifying self-reinforcing loops in patient behavior.

Our findings highlight that well-intentioned precautionary prescriptions can shape patients’ beliefs in unintended ways that potentially influence clinically inappropriate antibiotic demand. Supporting physicians in aligning their communication, and improving public understanding of self-limiting infections, is important to reduce inappropriate antibiotic use.

## Supplementary Material

ckaf146_Supplementary_Data

## Data Availability

The data, analysis code, and materials are publicly accessible (https://osf.io/rz2by/). The studies were preregistered (https://aspredicted.org/KQK_B7F and https://aspredicted.org/7jyh-44c9.pdf). Key points Precautionary antibiotic prescribing can lead patients to attribute their recovery to antibiotics. These beliefs increase patient expectations for antibiotics in future, unrelated illnesses. Explaining that many infections resolve without antibiotics helps correct these beliefs. Embedding such messages on self-limiting infections in both clinical encounters and wider public health communication could reduce inappropriate antibiotic use.
