# Supplementary material for: Recovery attributions and future expectations for antibiotics after precautionary prescribing
Source: Eur J Public Health. 2025 Aug 26;35(5):1020–5. doi: 10.1093/eurpub/ckaf146 (PMC12529262; doi:10.1093/eurpub/ckaf146)
Supplement: ckaf146_Supplementary_Data [file ckaf146_supplementary_data.docx]

**Supplement**

**Recovery attributions and future expectations for antibiotics after precautionary prescribing**

**Table S1**

**Overview of participant characteristics Study 1**

|  | **high base rate (N=82)** | **low base rate (N=85)** | **no base rate (N=85)** | **Overall (N=252)** |
| --- | --- | --- | --- | --- |
| **Age** |  |  |  |  |
| Mean (SD) | 44.1 (16.9) | 41.8 (13.0) | 41.8 (13.9) | 42.5 (14.6) |
| Median [Min, Max] | 41.5 [21.0, 83.0] | 39.0 [19.0, 77.0] | 39.0 [19.0, 78.0] | 40.0 [19.0, 83.0] |
| **Gender** |  |  |  |  |
| Man | 40 (48.8%) | 41 (48.2%) | 41 (48.2%) | 122 (48.4%) |
| Woman | 42 (51.2%) | 42 (49.4%) | 41 (48.2%) | 125 (49.6%) |
| Non-Binary | 0 (0%) | 2 (2.4%) | 0 (0%) | 2 (0.8%) |
| Other | 0 (0%) | 0 (0%) | 2 (2.4%) | 2 (0.8%) |
| Prefer not to say | 0 (0%) | 0 (0%) | 1 (1.2%) | 1 (0.4%) |
| **Education** |  |  |  |  |
| Some high school | 1 (1.2%) | 1 (1.2%) | 0 (0%) | 2 (0.8%) |
| High school diploma or equivalent | 11 (13.4%) | 19 (22.4%) | 15 (17.6%) | 45 (17.9%) |
| Some college, no degree | 23 (28.0%) | 19 (22.4%) | 18 (21.2%) | 60 (23.8%) |
| Associate's degree | 2 (2.4%) | 2 (2.4%) | 4 (4.7%) | 8 (3.2%) |
| Bachelor's degree | 33 (40.2%) | 23 (27.1%) | 26 (30.6%) | 82 (32.5%) |
| Master's degree | 9 (11.0%) | 16 (18.8%) | 18 (21.2%) | 43 (17.1%) |
| Doctorate | 3 (3.7%) | 5 (5.9%) | 3 (3.5%) | 11 (4.4%) |
| Other | 0 (0%) | 0 (0%) | 1 (1.2%) | 1 (0.4%) |

**Table S2**

**Overview of participant characteristics Study 2**

|  | **high base rate (N=1233)** | **no base rate (N=1215)** | **Overall (N=2448)** |
| --- | --- | --- | --- |
| **Age** |  |  |  |
| Mean (SD) | 41.5 (13.5) | 42.3 (13.9) | 41.9 (13.7) |
| Median [Min, Max] | 39.0 [18.0, 87.0] | 41.0 [18.0, 80.0] | 40.0 [18.0, 87.0] |
| **Gender** |  |  |  |
| Man | 654 (53.0%) | 597 (49.1%) | 1251 (51.1%) |
| Woman | 566 (45.9%) | 603 (49.6%) | 1169 (47.8%) |
| Non-Binary | 8 (0.6%) | 10 (0.8%) | 18 (0.7%) |
| Other | 1 (0.1%) | 0 (0%) | 1 (0.0%) |
| Prefer not to say | 4 (0.3%) | 5 (0.4%) | 9 (0.4%) |
| **Education** |  |  |  |
| Some high school | 29 (2.4%) | 18 (1.5%) | 47 (1.9%) |
| High school diploma or equivalent | 172 (13.9%) | 178 (14.7%) | 350 (14.3%) |
| Some college, no degree | 248 (20.1%) | 226 (18.6%) | 474 (19.4%) |
| Associate's degree | 37 (3.0%) | 40 (3.3%) | 77 (3.1%) |
| Bachelor's degree | 489 (39.7%) | 510 (42.0%) | 999 (40.8%) |
| Master's degree | 224 (18.2%) | 209 (17.2%) | 433 (17.7%) |
| Doctorate | 30 (2.4%) | 27 (2.2%) | 57 (2.3%) |
| Other | 4 (0.3%) | 7 (0.6%) | 11 (0.4%) |

**Sensitivity Analyses Study 2 (results without those that did not pass the encoding check)**

Participants generally attributed their recovery to the antibiotics. However, participants in the high base-rate condition attributed their recovery significantly less to antibiotics (*M* = 59.07, *SD* = 28.12) than those in the no base-rate (control) condition (*M* = 74.15, *SD* = 20.89), t(1848.9) = –13.78, p < .001, d = –0.61, 95% CI [–0.70, –0.52].

There was a small but significant positive correlation between recovery attribution and expectations of receiving antibiotics for a future illness, r = .11, p < .001. That is, the more strongly participants attributed their recovery to antibiotics, the more likely they were to expect antibiotics in a subsequent illness.

When analysed by condition, the correlation was r = .09 (p = .003) in the no base-rate condition and r = .15 (p < .001) in the high base-rate condition. However, as shown in Figure 2B, a one-tailed Fisher z-test revealed that this difference between correlations was not statistically significant, z = –1.47, p = .929.
